# A Radicular Cyst and an Aneurysmal Bone Cyst Converging in the Maxilla: A Concurrent Encounter

**DOI:** 10.7759/cureus.69232

**Published:** 2024-09-11

**Authors:** Thomson Mariadasan Dcruz, Shreyas H Gupte, Drishti Shah, Pearlcid Siroraj

**Affiliations:** 1 Oral and Maxillofacial Surgery, Dr. G.D. Pol Foundation`s Y.M.T. Dental College and Hospital, Navi Mumbai, IND; 2 Oral and Maxillofacial Surgery, Sri Ramachandra Institute of Higher Education and Research, Chennai, IND

**Keywords:** aneurysmal bone cyst, case report, maxilla, pseudocyst, radicular cyst

## Abstract

We present a unique case of a patient in early adolescence presenting with swelling in the upper left back region of the jaw, attributed to a failed root canal treatment of the upper left first molar. Clinical and radiographic assessments led to a preliminary diagnosis of a radicular cyst. Surgical enucleation revealed unexpected findings, with a steady ooze of blood encountered from one side of the lesion and the retrieval of an epithelial lining from the other side. Histologic examination confirmed the presence of both a radicular cyst and a secondary aneurysmal bone cyst (ABC). To the best of our knowledge, this is the first reported case of an ABC of the maxilla coexisting with a radicular cyst. This case underscores the importance of thorough evaluation and histological examination in cases of suspected cystic lesions, as well as the potential for unexpected findings and coexisting pathologies.

## Introduction

The term aneurysmal bone cyst (ABC) is misleading as it is not lined by the epithelium and it is not a true cyst. An ABC is a locally destructive and rapidly expanding benign lesion of the bone consisting of blood-filled spaces. [1] The occurrence of this lesion in the jaws is rare, but when it does occur, the mandible is more commonly affected than the maxilla (2:1) [2-5]. Only 1.8% of ABCs usually occur in the jaw bones [6]. This rare occurrence of ABCs was first recognized by Jaffe and Lichtenstein as a clinic-pathological entity in 1942 [7]. Bernier and Bhaskar described the first case of the ABC in the jaws in 1958. Only 22 cases of ABCs located in the maxilla have been reported so far justifying the rare occurrence of this multicystic lesion. ABCs can be classified into three types: Conventional or vascular type which usually presents as an expansile, rapidly growing destructive lesion causing cortical perforation and soft tissue invasion. The solid type reveals a small asymptomatic lesion first noticed as a radiolucency on a routine radiograph or as a small swelling clinically. The third form is a mixed variant exhibiting features of both the vascular and solid types. A diagnosis of a radicular cyst is usually considered when the lesion is associated with a grossly decayed non-vital tooth. We report an unusual case of a solid type of ABC of maxilla coexisting with a radicular cyst creating a diagnostic dilemma and suggesting potential for ABCs to be co-existing with pre-existing radicular cysts. 

## Case presentation

A 15-year-old female patient reported to our clinic with a complaint of pain and swelling in the upper left back tooth region of the jaw for the past one month.

On intra-oral examination, a diffuse swelling, in the maxillary left buccal vestibule, extending antero-posteriorly from distal of 25 to mesial of 27 and supero-inferiorly from the gingival margin up to the depth of the buccal vestibule, approximately 2 X 2 cm in size was present. The overlying mucosa appeared normal without any signs of bleeding or pus discharge. There was obliteration of the buccal vestibule and expansion of the buccal cortical plate. On palpation, the swelling was non-tender, non-fluctuant, non-pulsatile, and firm in consistency. Hard tissue examination revealed a failed root canal treated 26. The swelling appeared to be insignificant extra-orally. 

**Figure 1:**
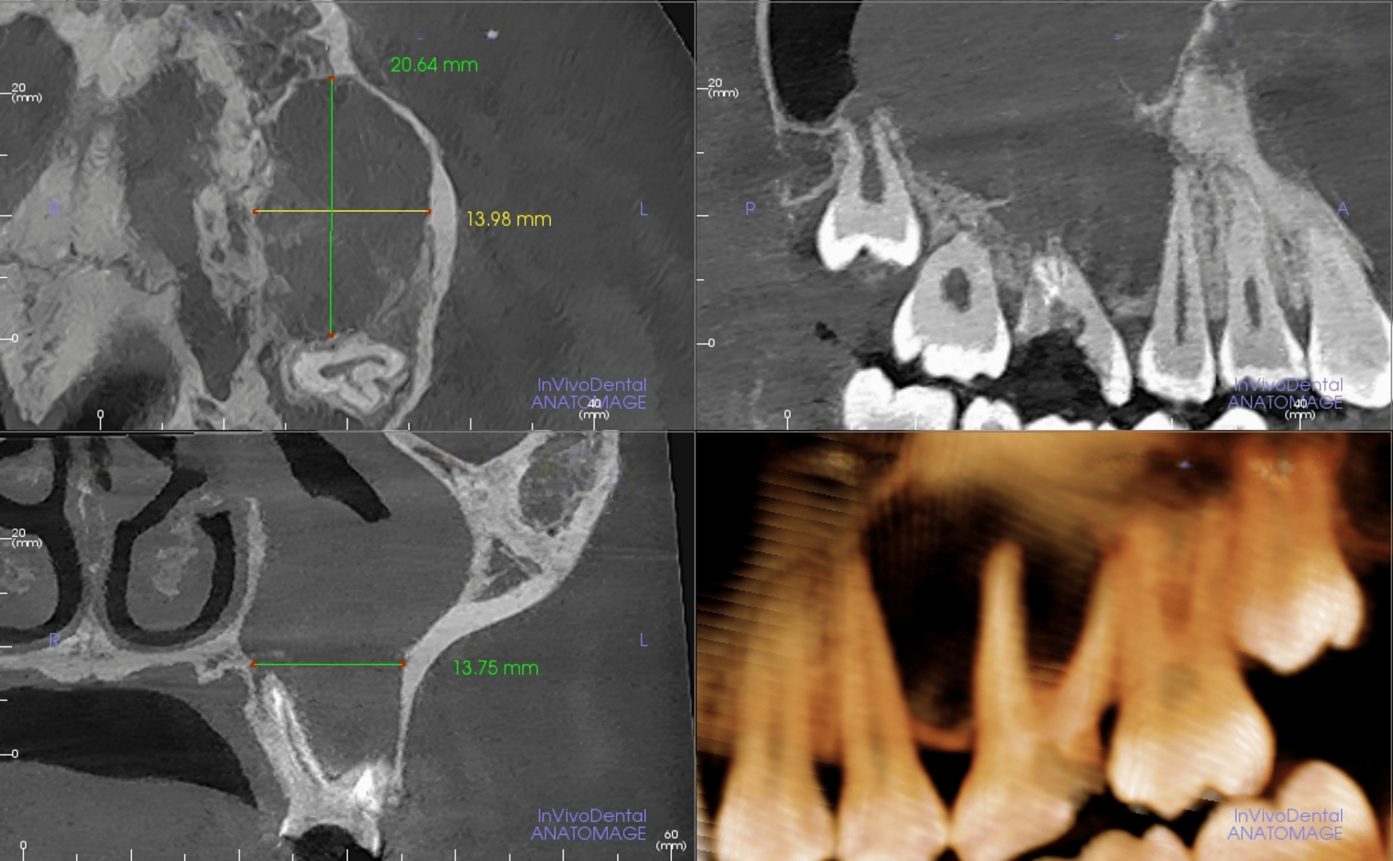
Multiplanar reconstruction of CBCT showing a hypodense lesion in the left maxillary region

Cone beam computed tomography (CBCT) cross-sectional images showed a large destructive lesion seen in the left maxillary region extending from the distal aspect of tooth 25 up to the mesial aspect of 27 antero-posteriorly (Figure 1). Supero-inferiorly, the lesion extends from the alveolar crest into the left maxillary antral cavity. The margins are slightly irregular and partially corticated.

Considering the clinical and radiographic findings, a provisional diagnosis of an infected radicular cyst was considered. Based on the clinical and radiographic findings, a differential diagnosis of a radicular cyst, an odontogenic keratocyst, an adenomatoid odontogenic tumor, and unicystic ameloblastoma was considered.

**Figure 2:**
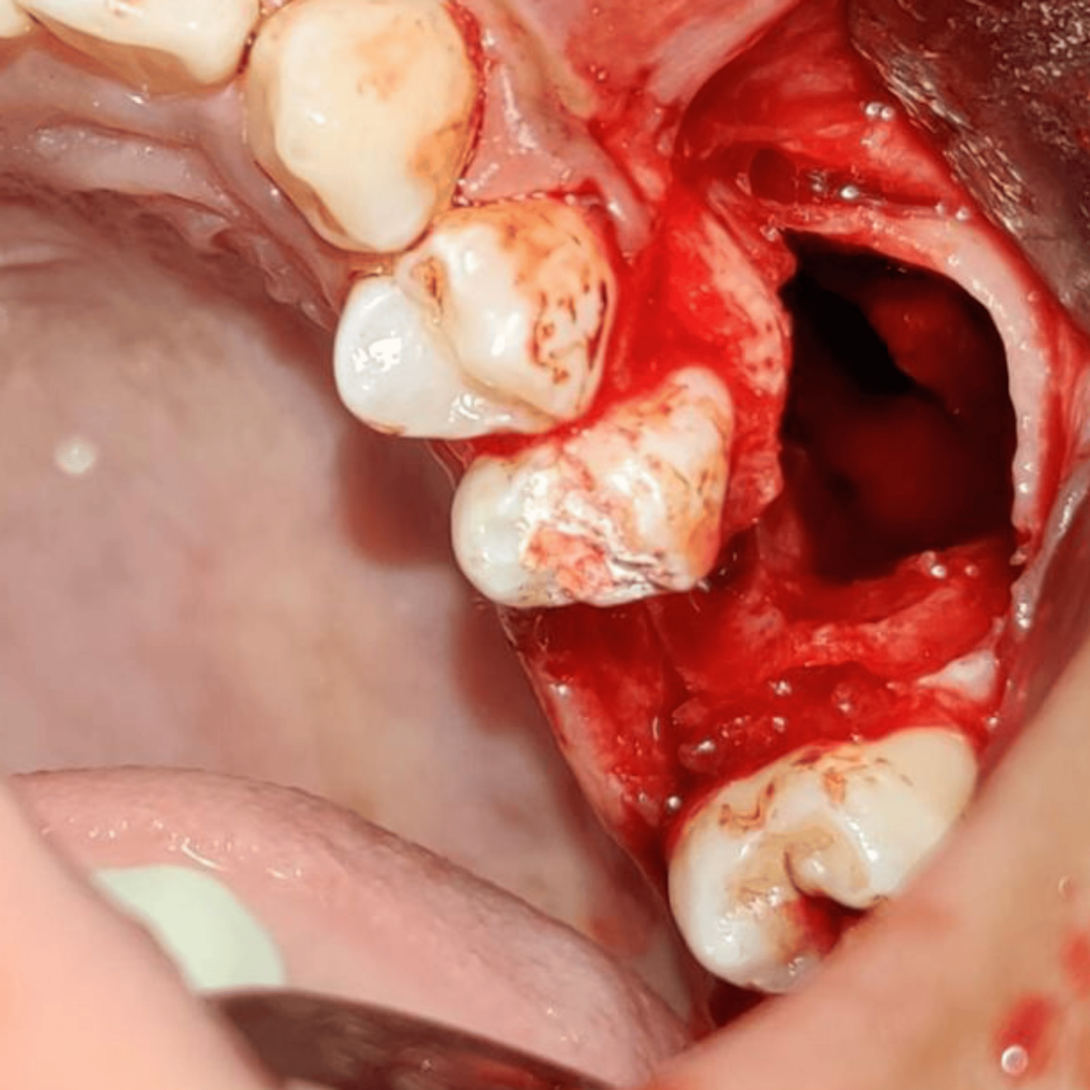
Clinical image showing cystic cavity following extraction of the maxillary left first molar

Enucleation and curettage of the lesion were planned under local anesthesia, followed by extraction of tooth 26. A full-thickness mucoperiosteal flap was raised from the mesial of tooth 25 to the mesial of tooth 27. Upon deroofing, the cystic lesion was exposed, demonstrating close proximity to the maxillary sinus. Upon entry into the lesion, a steady ooze of blood was encountered, which was atypical for a radicular cyst (Figure 2). The lesion appeared reminiscent of a blood-soaked sponge and was subsequently sent for histopathological examination. Notably, epithelial lining was observed in some parts of the lesion. Bone grafting was done following enucleation and curettage of the cystic lesion. 

**Figure 3:**
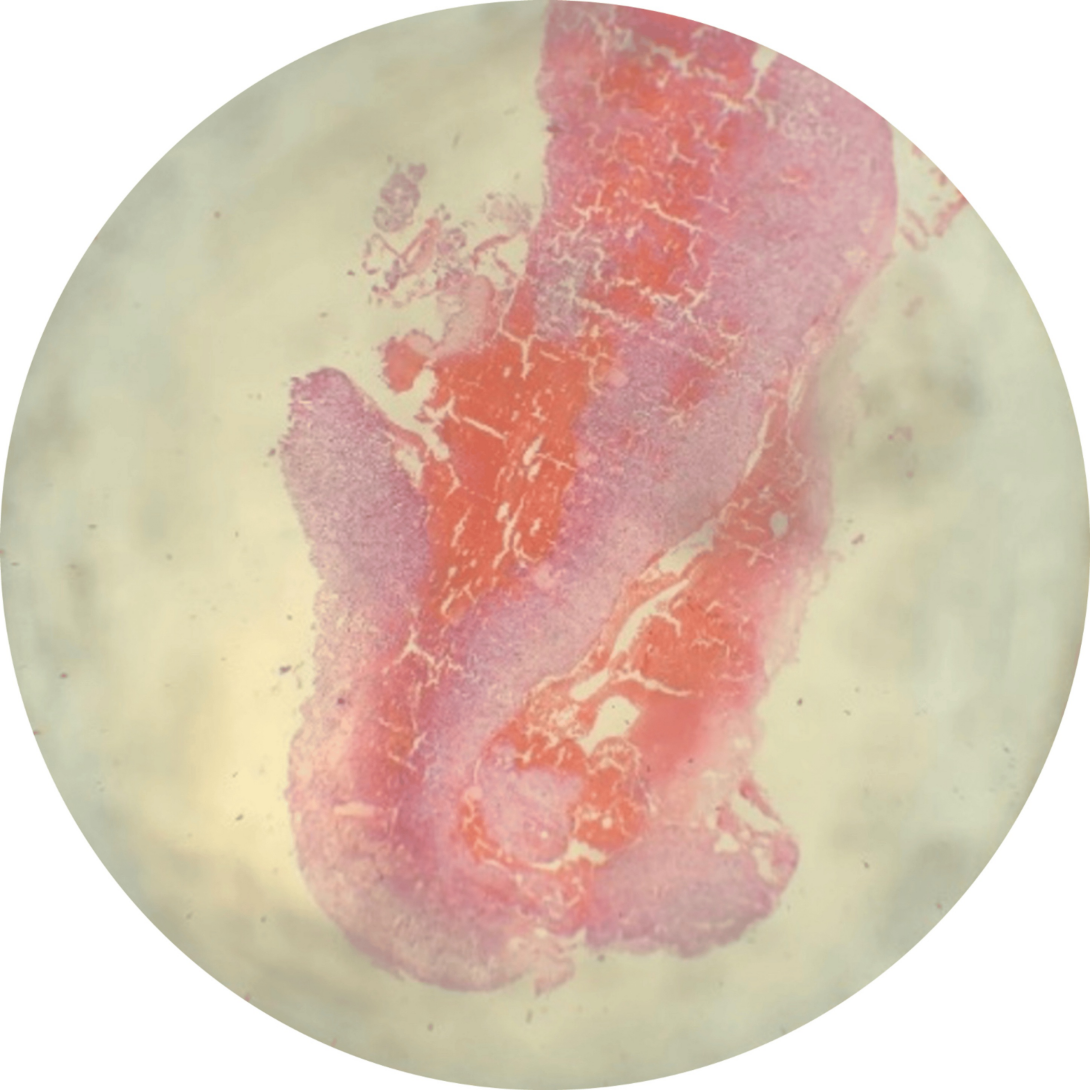
H & E stained sections showing the characteristic features of the aneurysmal bone cyst

**Figure 4:**
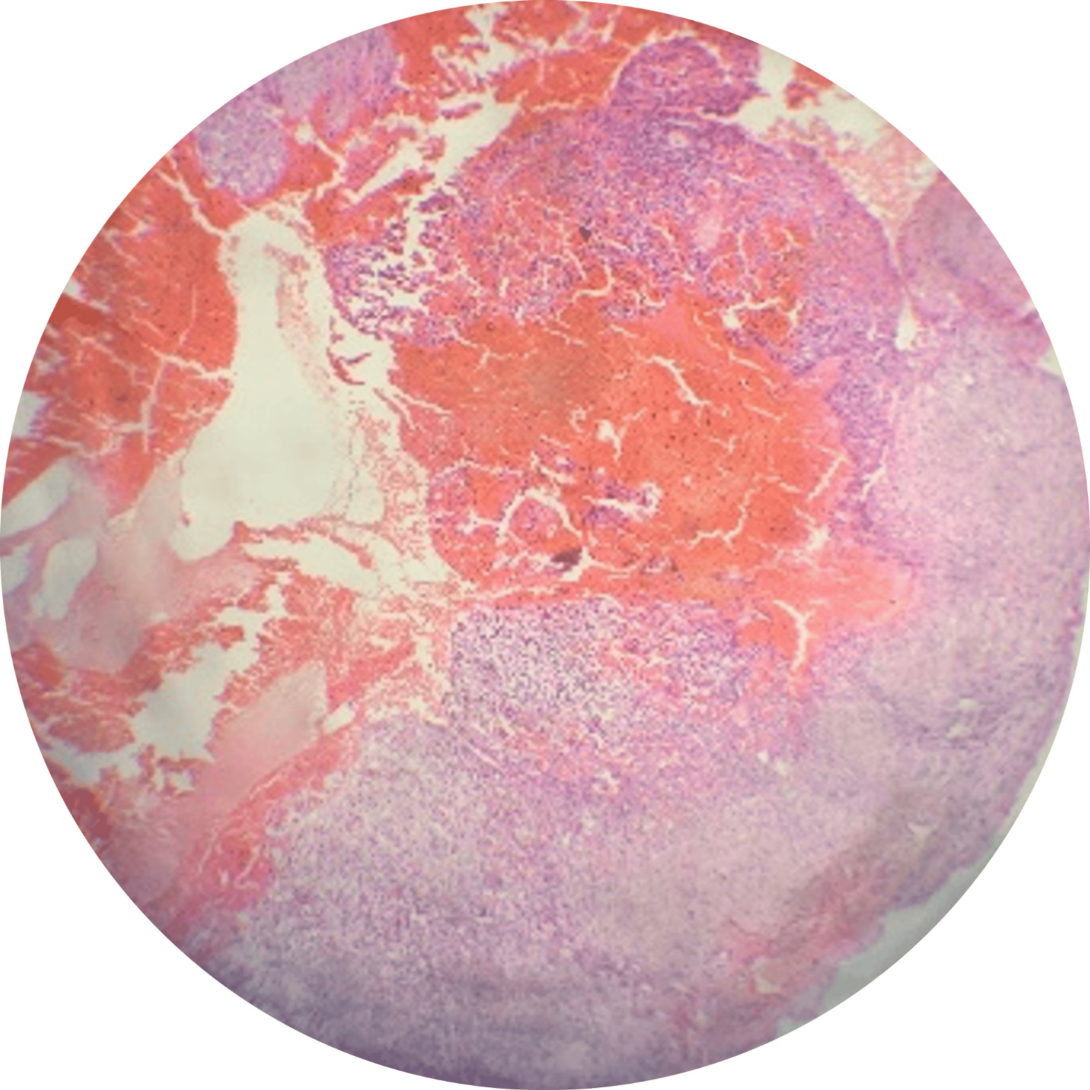
H & E stained sections showing the characteristic features of the aneurysmal bone cyst

Histopathological examination revealed multiple blood-filled spaces within the lesion, accompanied by stroma consisting of spindle-shaped cells, multinucleate giant cells, and areas of osteoid with prominent osteoblastic rimming (Figure 3 and Figure 4).

**Figure 5:**
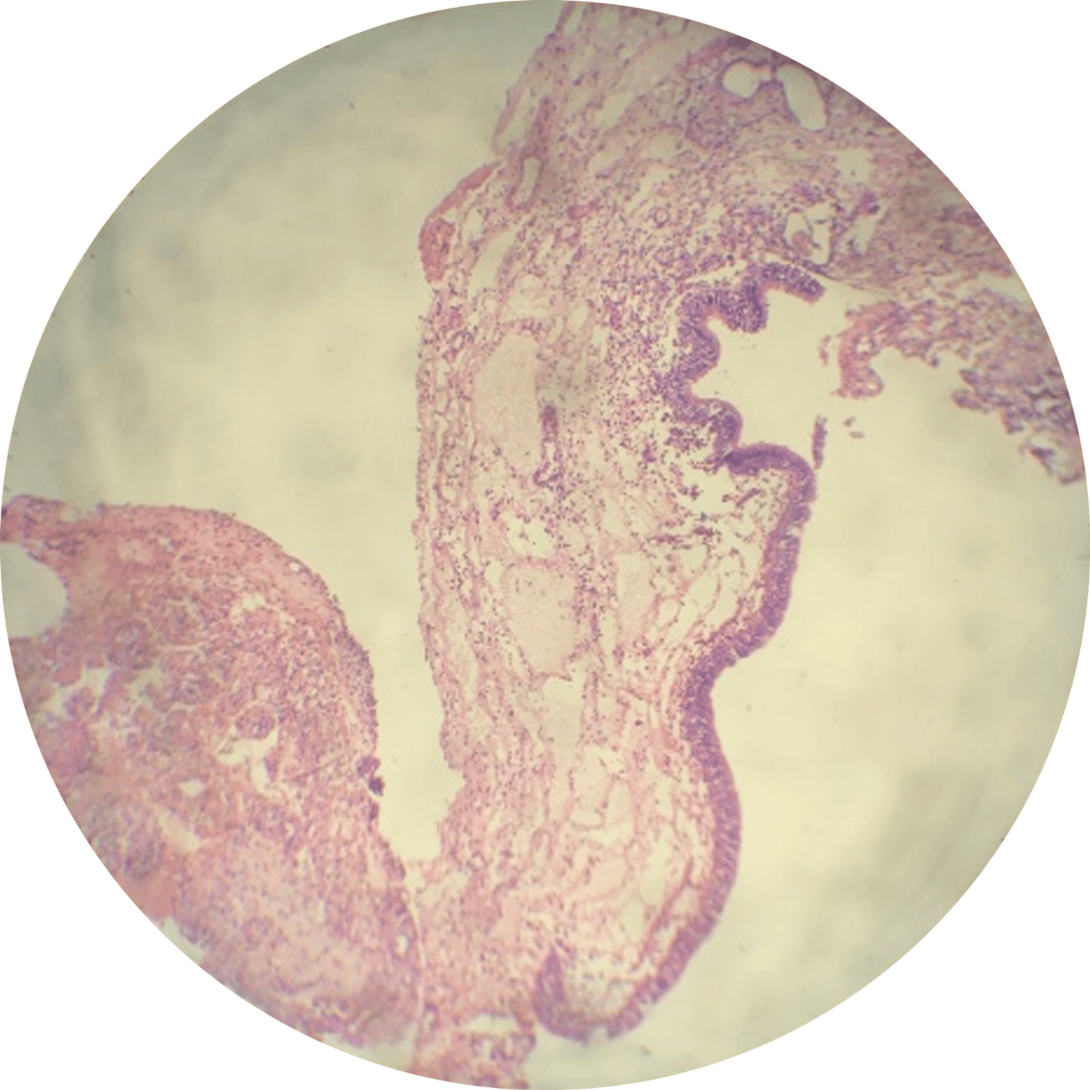
H & E stained section showing epithelial lining characteristic of the radicular cyst

The histopathological report confirmed the diagnosis of an ABC associated with an antecedent radicular cyst (Figure 5).

The post-operative period was unremarkable, and the patient is on a regular follow-up with no clinical evidence of recurrence noted one year later.

## Discussion

ABCs are rare non-epithelialized pseudocysts of the jaws. The term ABC is a misnomer since it is neither an aneurysm nor a cyst. First reported by Bernier and Bhaskar in the facial bones in the year 1958, the ABC remains uncommon in the facial bones. This may be because the lesion is most common in those areas of the skeleton where there is both a relatively high venous blood pressure and a high marrow content but is relatively rare in the skull because of the reduced venous blood pressure [7]. This case report shows a rare occurrence in the posterior maxilla. ABCs are more common in the first three decades with a peak from 10 to 20 years [8,9]. This is consistent with the findings of the present case.

The etiology of ABCs is controversial and debatable. Circulatory disturbance and altered hemodynamics resulted in dilatation and congestion of the vascular bed causing resorption of the spongy bone and cortex which was further replaced by fibrous connective tissue and osteoid [5]. This theory was suggested by Jaffe and Lichtenstein. Levy et al. and Tillman et al. reported that the development of ABCs was related to a history of trauma [10,11]. There was no such history of trauma in the present case report [12]. Biesecker et al. suggested that an arteriovenous malformation is formed as a bone lesion, which results in an increased hemodynamic force that leads to a secondary reactive lesion which eventually becomes an ABC [6]. ABCs have both primary and secondary variants depending on their association with any pre-existing pathology [13].

Radiological characteristics are similar between ABCs and a few other tumors of the face. ABCs transform through four radiographic stages namely the initial stage, active growth phase, stabilization phase, and healing stage [14]. Initially, the lesion has a marked area of well-defined osteolysis followed by rapid growth of the lesion which leads to gradual lysis of bone [15]. The bony destructions lead to the characteristic “blown out” appearance on a radiograph. The period of stabilization is characterized by maturation of surrounding bone which results in a “soap bubble appearance” [16]. Calcification and ossification are the characteristic features of the healing phase, and the lesion eventually transforms into a dense bony mass [17]. The present case report demonstrated the initial stages of radiographic appearance. Regezi and Sciubba in 1993 and Goaz and White in 1994 claimed that the margins of the lesion were slightly irregular which was a consistent finding in our case report [18-20].

Treatment is determined by the nature of the associated lesion. Although there are many treatment options available, surgical excision and curettage remain the gold standard due to the benign nature of this lesion. During surgery and upon removal of the bone shell, the lesion exhibited welling of dark venous blood, which was observed in our case and was manageable [21]. Few authors have recommended supplementary curettage with cryotherapy and embolization [22]. The recurrence rate following curettage is 21-50% in the case of jaw ABCs [4]. The treatment of ABCs can vary from simple curettage to en bloc resection, depending on the age of the patient, the location and size of the lesion, and the extent of bony destruction.

In this case, the presence of an endodontically treated non-vital tooth and the findings of CBCT lead us to provide a provisional diagnosis of a radicular cyst. However, histopathological evaluation confirmed the diagnosis of an ABC. Preoperative diagnosis of the ABC is difficult due to its similarity to various other lesions.

Recognition of a coexistence of ABCs and a precursor primary lesion at the time of surgery is crucial not only in terms of diagnostic accuracy but also for the assessment of the difficulties that can be encountered during complete removal of the lesion and thus, the possible chance for recurrence of the lesion.

ABCs do not have a fixed radiological appearance and should be included in the differential diagnosis of radiolucency of the jaws and any mixed radiopaque-radiolucent lesion.

Each lesion of the jaws has characteristic radiographic and clinical presentations that aid clinicians in providing an accurate diagnosis. However, similar clinical manifestations, inadequate definition of specific characteristics, inconsistent signs and symptoms in patients, and varied clinical manifestations of a single lesion lead to errors in clinical diagnosis. Thus, clinical examination, radiographic investigation, aspiration cytology, and incisional biopsy are crucial for the diagnosis of ABCs.

## Conclusions

This case report highlights the diagnostic challenges and successful management of a rare coexistence of a radicular cyst with an ABC in the maxilla. It emphasizes the importance of comprehensive evaluation, including radiographic imaging and histopathological examination, in guiding treatment decisions for such complex lesions. Radiographic investigations are suggestive but not diagnostic for ABCs. The radiologic and clinical presentation of ABCs is extremely variable and a great emphasis is placed on histopathologic examination for the diagnosis.

An ABC occurs as a result of secondary changes in a pre-existing, incipient, and slow-growing lesion. The propensity of formation of ABCs secondary to a radicular cyst may follow a familiar phenomenon of disturbed hemodynamic alteration corresponding to the growth of the radicular cyst.
